# Improvement of Lidocaine Local Anesthetic Action Using *Lallemantia royleana* Seed Mucilage as an Excipient

**Published:** 2014

**Authors:** Rabi Atabaki, Majid Hassanpour-ezatti

**Affiliations:** Biology Department, School of Sciences, Shahed University, Tehran, Iran.

**Keywords:** Lidocaine, Plant mucilage, Anesthesia, Rats, Acute pain, Topical administration

## Abstract

*Lallemantia royleana* (Balangu) is a well known Iranian medicinal plant that its seed mucilage has many applications in modern pharmacology. Plant mucilage traditionally was used as a gel supplement, and natural matrix for sustained release of drugs. But it seems that these compounds are not a simple additive and also have many undiscovered pharmacological properties.

In this research, the anesthetic action of gel prepared from Balangu mucilage alone and its mixture with lidocaine hydrochloride are compared with the effect of commercial 2% lidocaine gel by rat tail flick test.

Mucilage of Balangu seed alone showed analgesic effect. Duration and potency of anesthesia induced by gel containing mucilage alone (0.01 g/mL) were identical to commercial 2% lidocaine gel. But, local anesthetic potency and duration of gel made from 2% lidocaine-mucilage gel mixture was significantly higher than commercial 2% lidocaine gel.

The gel prepared from mucilage causes a good analgesia with unknown mechanism. Besides, mixture of Balangu mucilage prepared gel with lidocaine improves lidocaine anesthesia. The increase in potency of lidocaine action results from mucilage dermal penetration enhancing effects; and longer anesthetic duration of this mixture are related to the capability of mucilage based gel for sustained drug release.

## Introduction

Compounds generating local anesthesia are widely used in surgery, treatment of chronic pains, post-surgery pain relief and have vast majority of other medical applications (1,2). Lidocaine commercial gel as a local anesthetic was generally used for creation of topical anesthesia and reduction of local pains. Short duration of action and low anesthetic intensity are considered as disadvantages of topical form of lidocaine (3,4). Due to the different barriers in the epidermis, induction of topical anesthesia by local prescribe drugs on healthy and intact skin is not easy (5). Indeed, lidocaine incomplete diffusion from the skin is proposed as an explanation for its low potency (6), which originates from its low skin penetrability (7). This problem was solved by adding some pharmacological excipients to lidocaine that were named penetration enhancers. Terpenes are a group of herbal active compounds that exhibit skin penetrating properties and it has been reported that these compounds can increase local anesthetic action of lidocaine gel (8). For instance, adding α-terpineol to lidocaine gel could increase the local anesthetic effects of lidocaine gels (6). Previously, it was shown that the mixture of lidocaine with a herbal mucilage can increase duration of its local anesthesia (9). On the other way, herbal mucilage as a type of polysaccharide is non-toxic, has high chemical and physical stability compared to the synthetic forms and after dissolving in water easily makes a gel (10). Recently, the gel prepared from plant mucilages has been used as a compound for sustained drug release in drug delivery systems (9). Currently, plant mucilage is used by researchers as a pharmaceutical excipient (11). Other attractive abilities of mucilage for pharmaceutical applications are using them as thickening, stabilizing agents, additive, release retardants, binding, emulsifying, gelling, suspending, and disintegrating in tablets (12, 13). Indeed, the number of successful usages of herbal mucilage as supplement for sustained-drug release is growing now (14,15). The plant *Lallemantia royleana* (Labiatae) with the conventional name “Balangu” is a plant that growth in Iran (16). Seeds of this plant produce lots of mucilage after maceration in water. This plant grows in Iran and nearly all parts of Middle East (17). The dry mucilage of Balangu after dissolution in water generates a white and clear gel (18). The use of this plant seed has long historical background in human life (19), and the presence of terpenic compounds in seed of this family is reported previously (17). Also, gel prepared from Balangu seed mucilage is prescribed for treatment of some digestive problems and reduction of abscess (20). Their anti-inflammatory effects are also reported (21). The muco-adhesive properties of Balangu mucilage are better than many other pharmaceutical supplements like chitosan, Carbopol 934, hydroxypropyl, and methylcellulose, (22). So, this mucilage may be a good candidate as an excipient gel for topical anesthetic drugs. It is reported that the routine adjuvant added to lidocaine produces side effects like paleness or redness on skin surface of users. These side effects are consequences of rapid release of lidocaine and its contracting effect on skin peripheral vessels (23). So, adding a natural compound with release retarding property to lidocaine may control lidocaine fast release from gel and also reduce its undesirable effects compared to commercial additives. 2% lidocaine hydrochloride gel is the conventional form of this gel (24). Some experts tried to increase anesthetic potency of lidocaine gel by increasing its dose for using this gel for some especial conditions (25). In an attempt to solve some of the problems mentioned above, the gel made from *Lallemantia royleana* mucilage was used as an excipient for preparing topical lidocaine gel. Then, the analgesic potency and duration of this gel mixture with %2 lidocaine hydrochloride, was evaluated after topical application on tail of adult male rats with tail flick method proposed by Jin & Shin (26).

## Experimental

*Microwave assisted mucilage extracting method*

*Lallemantia royleana* plant seed was purchased from a medicinal plant store and its genus and species was confirmed by a plant biosystematician. For mucilage extraction, dried and cleaned Balangu seed (2.5 g) was soaked in 100 mL distilled water for 24 hours. Pure Balangu seeds mucilage free from seed residues was extracted, dried, and sterilized by a new invented method using microwave oven (13,14). In practice, macerated seeds were spread in the bottom of a petteri dish (to the thickness of one seed) and dried slowly in a microwave oven (Panasonic, IRAN) with 220 watt power for 55 minutes. Then, the dried mucilage was **scraped** with a **scalpel** blade, powdered and kept in a sterile vial at room temperature until experiment day. During experiment, dried mucilage powder (0.01 g) was dissolved in distilled water (1 mL) and completely mixed until a clear and bright gel was prepared. 

*Preparation mixture of mucilage-lidocaine hydrochloride*

Lidocaine hydrochloride powder taken as a gift from Darou Pakhsh Company (Tehran, IRAN) and the gel prepared from the extracted mucilage was completely mixed with 2% lidocaine hydrochloride powder. The mixture was kept at 4 °C in the refrigerator for 48 hours and then used. The utilized mixture should be clear, without any precipitation and its color shouldn’t change. 

*Tail flick experiments*

The male Wistar rats (240 to 300 g) were obtained and divided into these groups: 1-control group, 2-mucilage treated group, 3-commercial 2% lidocaine gel treated and 4- mixture of mucilage gel with 2% lidocaine group. The number of rats in each group was 5. The tail flick latency was measured in all the groups by applying thermal light beam to the tail of each rat based on Jin & Shin procedure (26). The tail flick latency of rats was measured by a domestic automated tail flick analgesiometer device equipped with a projector lamp manufactured by Borj Sanat (http://borjsanat.ir). Beam intensity was adjusted to level that give a tail-flick latency of 5-8 sec in control animals. A 20 second cut-off time was used to avoid thermal injury. Before topical application of each medication, two tail flick experiments were taken from each rat. Its average was considered as a baseline latency of rats. Then, according to the experimental groups; 250 (mg) of mucilage alone, 2% lidocaine commercial gel or mucilage-lidocaine hydrochloride mixture was applied on 2⁄3 distal part of each rat tail and the rat was tested in response to painful stimulation every five minutes for 55 minutes. In control groups, rat tails were treated topically only with 250 microliter distilled water.

*Statistical analysis of data*

The average of tail flick latency of each rat was calculated during 55 minute with 5 minute interval and compared between groups by two-way ANOVA (p<0.05) and Bonferroni post test.

## Results

**Figure 1:**
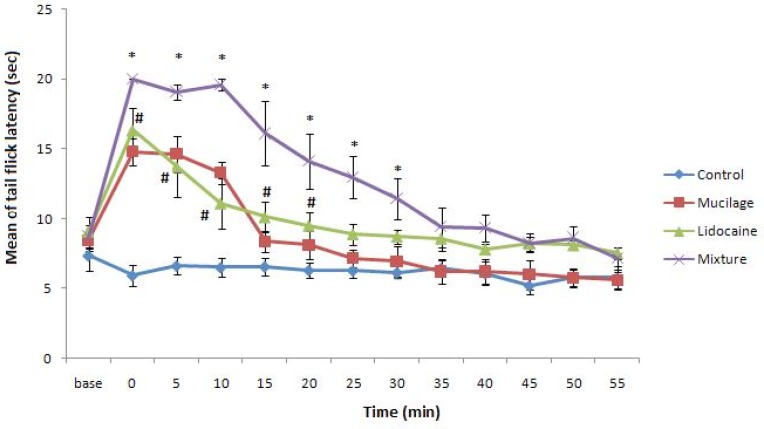
Tail flick latency (mean ±SEM) of rats was measured before (base) and after each treatment. Rats groups: Control, topically treated with distilled water; Commercial lidocaine, treated with 2% commercial lidocaine gel; Mucilage, treated with mucilage alone; Mixture, treated with mucilage+2%lidocaine (Two-way ANOVA and Bonferroni post test). * p<0.05 Mixture group v.s. Commercial lidocaine group and # p<0.05 Mucilage or Commercial lidocaine groups v.s. control.”

Our prepared gel was white and clear and lidocaine powder was easily mixed with it without any change in mucilage quality. The mixture of gel and lidocaine before use was kept in refrigerator up to 48 hours for complete mixing of them and no especial change was observed in its appearance during this period. Tail flick latency obtained from control and experimental groups were shown in Figure 1. Our data indicates topical applying of mucilage alone on rat tail led to increase in rat tail flick latency with the same potency of rat that was treated with commercial 2% lidocaine gel. Also, the duration of anesthesia in rats that topically were treated with mucilage alone was same as rats that were treated with commercial 2% lidocaine gel (Figure 1). But tail flick latency of rats treated with 2% lidocaine-mucilage gel mixture significantly increased and stayed for about 35 minutes in this situation in comparison with other experimental groups. 

## Discussion and Conclusion

Our findings showed the lidocaine-balangu mucilage gel mixture kept in (4 °C) refrigerator were stable even after long time. The long duration of Balangu mucilage gel physical stability was confirmed for other application, too (27). Previous experiments demonstrated that mixture of different compounds with this gel doesn’t change the molecular structure of gel and does not disrupt its rheological properties (27). So, the gel prepared from Balangu mucilage could be considered as a good drug carrying matrix for many pharmaceutical compounds. Also, the gel had shown suspension property in combination with many chemical substances (22). Our results proved that the local application of the Balangu gel alone can increase acute pain threshold and its anesthetic duration is same potency as commercial 2% lidocaine gel. Also, the local application of Aloe Vera plant mucilage gel in animals could reduce pain threshold (28). The reactive oxygen species (ROS) compounds acted as pain mediators in many different tissues and can stimulate nociceptors (29). The role of ROS in induction of acute pain was confirmed by different animal pain models (30). Also, it was reported that acute analgesia effect of morphine was mediated through inhibition of ROS production (31). The antioxidant activity and ROS reducing effect of some herbal mucilage were demonstrated (32, 33). For example, mucilages extracted from three different kinds of Yam plants have shown antioxidant and radical scavenger properties (34). By paying attention to chemical similarity of mucilages, the Balangu seed mucilage analgesic effect probably was mediated through control of ROS compound production. The upper layer of skin or stratum corneum is the main barrier in the way of dermal application of drugs and rate limiting step in absorption of topical drugs (35). In addition, mucilage based gel has a good dermal penetration power by itself and so can potentiate dermal penetration rate of drugs as a pharmaceutical supplement. It has been shown that herbal mucilage has high skin penetrating quality; for example, adding Aloe Vera mucilage to some drugs can increase dermal absorption of them. The mechanism of their action has not been identified partly (36). As it was mentioned earlier, lidocaine is incompletely diffused from skin (6), and improperly passes from healthy skin (7). Our results showed the anesthetic potency of mucilage-lidocaine mixture was higher than commercial available gel of lidocaine. The polysaccharide constitutes the main part of *Lallemantia royleana* mucilage gel similar to other mucilage such as Aloe Vera mucilage (37). Thus, the probable presence of plant polysaccharides can increase dermal penetration and improve dermal absorption of lidocaine and enhance its pharmacological potency. The presence of trepenoid compounds in the mucilage of Balangu gel can help as other mechanisms that increase its dermal penetration (8). Data gathered from *in-vivo* and *in-vitro* research denoted the enhancement of topical drugs effect after mixing with a gel matrix prepared from plant mucilage (38). It was denoted that the commercial preparation of lidocaine contains excipient which increase the rate of its absorption from skin (39, 40), and these excipient compounds have sugar based structures such as alkyldisiloxane or glucopyranosyl. These chemical groups also were found in the chemical structure of mucilages (41). In support of our observation, it was reported that Aloe Vera mucilage alone has analgesic action and can increase dermal penetration of drugs and be added as supplement to them (42-44). Our results show that the analgesic duration of 2% lidocaine hydrochloride-balangu gel mixture was longer than its commercial form. In explanation of present research, we proposed that mucilage prepared gel works as sustain releasing matrix. Currently, the capability of mucilages as slow releasing matrices was proven (45). For instance, this property of mucilage caused scientists use them in structure of drugs that are prepared for treatment of wounds, diagnosis or treatment of cancer, prevention and treatment of bacterial and viral diseases (46). This character of herbal mucilage also changes it as ideal matrix for topical (15). Finally, commercial 2% lidocaine gel in some practical application has shown low therapeutic potency. Thus scientists increased lidocaine dosage for use in these situations (6,47-49). But, our findings in this research about enhancement of lidocaine anesthetic potency in mixture with Balangu gel can provide a suitable natural excipient for improvement of lidocaine anesthesia potency beside its effect on duration of lidocaine. 
